# Extra Oxygen Leads to Bubble Trouble: Portal Vein Gas Embolism from 3% Hydrogen Peroxide Ingestion

**DOI:** 10.7759/cureus.2136

**Published:** 2018-02-01

**Authors:** Joowhan Sung, Francesca Cossarini, Leonidas Palaiodimos, Benjamin Benson, Mimoza Meholli

**Affiliations:** 1 Department of Medicine, Albert Einstein College of Medicine/ Jacobi Medical Center; 2 Department of Radiology, Albert Einstein College of Medicine/ Jacobi Medical Center

**Keywords:** hydrogen peroxide poisoning, portal vein gas embolism, low concentration hydrogen peroxide

## Abstract

Hydrogen peroxide ingestion can cause gastric irritation and gas embolism; however, most reported cases are from the highly concentrated (≥35%) solution used in the industry and data on household-used 3% solution ingestion is scarce. We report a case of a portal vein gas embolism after ingestion of 3% hydrogen peroxide. The patient was managed conservatively with antacids and improved in 48 hours. Endoscopy and hyperbaric treatment were considered but not pursued. This is the fifth reported case of gas embolism after 3% hydrogen peroxide ingestion and stands in line with other reports where the patients improved with conservative management.

## Introduction

Hydrogen peroxide is an oxidizing agent used as a disinfectant in the household, for wound irrigation and instrument sterilization in medicine, and as a bleaching agent in manufacture. Accidental exposure to hydrogen peroxide is usually benign, however, ingestion of large quantities or high concentration can cause damage to the gastrointestinal tract and oxygen generated from catabolism could cause gas embolism, including portal vein and cerebral embolism [1-2]. Gas embolism has been reported in patients with concentrated (≥35%) hydrogen peroxide ingestion, however, cases caused by 3% concentration are rare [2-3]. We present a case of portal vein gas embolism caused by the accidental ingestion of hydrogen peroxide 3% solution.

## Case presentation

On a hot summer day in New York City, a 46-year-old male with a history of diabetes presented to the emergency department with sudden onset epigastric pain, nausea, and repetitive vomiting. His symptoms started soon after the accidental ingestion of 350 cc of 3% hydrogen peroxide solution, which was mistakenly taken as water by the patient. The patient presented to the emergency room approximately 14 hours after the ingestion. By that time, he had already vomited 10 times, including three episodes of dark bloody vomitus.

**Figure 1:**
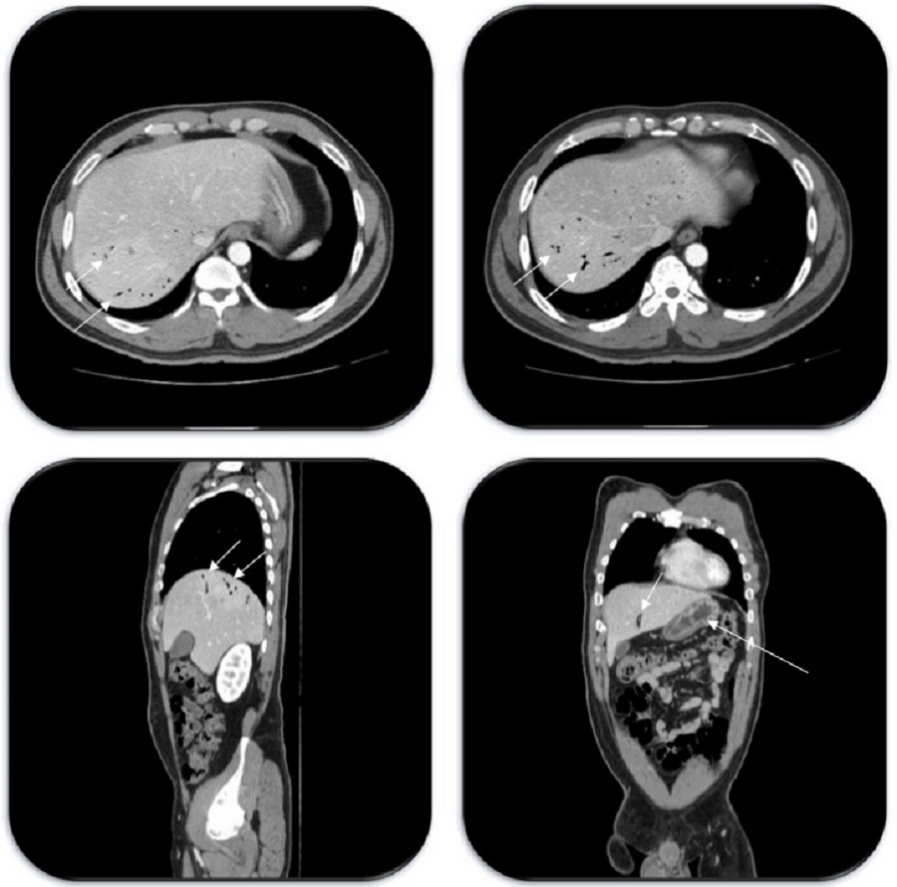
Axial (upper two images), sagittal (left lower image), and coronal (right lower image) abdominal CT scan images showing diffuse portal venous gas (short arrows) and gastric wall thickening (long arrow) up to 1 cm CT: computed tomography

On examination, he was afebrile and his abdomen was soft but with mild tenderness in the right upper and left upper quadrants. Laboratory investigations, including liver function tests, were unremarkable except for a mildly elevated total bilirubin of 1.4 mg/dL. A computed tomography (CT) scan of the abdomen and pelvis was performed, which revealed gastric wall thickening and diffusely scattered gas in the hepatic portal system (Figure 1). The patient was treated conservatively with intravenous hydration and antacids and was placed in the Trendelenburg position. Hyperbaric treatment was considered; however, it was not pursued as the patient’s symptoms were improving. Endoscopy was not pursued. Our patient’s symptoms improved over the following 48 hours and the patient was subsequently discharged.

## Discussion

Hydrogen peroxide is used at a wide range of concentrations (3% to 90%) [4]. Low concentration (3%) is used in the household and high concentration (35%) is used in the industry. It is caustic for human tissues and can cause damage if inhaled, ingested, or on contact with the skin. Inhalation can cause a spectrum of damage, from mild coughing to severe pulmonary edema, while skin contact leads to inflammation, blistering, and skin damage. Ingestion leads to irritation and inflammation of the gastrointestinal tract, resulting in nausea, vomiting, and sometimes hematemesis from hemorrhagic gastritis. Gas embolism, the most serious consequences, has mostly been reported after the ingestion of high concentration (35%) hydrogen peroxide [2]. Low concentration (3%) hydrogen peroxide ingestion is usually considered to be benign [3]. Our literature search revealed that only four other cases of portal vein embolism after the ingestion of 3% hydrogen peroxide have been described, making this case the fifth [5-8].

The mechanisms through which hydrogen peroxide causes tissue damage are corrosion, lipid peroxidation, and oxygen formation. Cells are protected from the oxidative damage of hydrogen peroxide by catalase, an enzyme present in the peroxisomes of nearly all aerobic cells, which catalyzes the decomposition of hydrogen peroxide to oxygen and water (2 H_2_O_2_ → 2 H_2_O + O_2_) [9]. However, the formation of large amounts of oxygen can have serious consequences. If a large volume of oxygen accumulates in a hollow cavity, such as the stomach, this can lead to distention, resulting in significant abdominal pain and possible rupture. In addition, oxygen is freely exchanged with the capillaries and if the amount of oxygen produced exceeds its maximum solubility, gas embolization may occur. Portal vein and systemic embolic events, including brain infarction due to gas embolism following hydrogen peroxide ingestion have been reported [1,9].

Since hydrogen peroxide is colorless and odorless, it can be easily mistaken for water, and it is important to maintain awareness of its toxicity even at low concentrations (3%). Our patient ingested a relatively large amount (350 cc) of 3% hydrogen peroxide, thus explaining the significant symptoms experienced and the formation of large amounts of oxygen leading to gas embolism into the portal vein system. When 1 mL of 3% hydrogen peroxide is catalyzed, it produces 10 mL of oxygen gas under normal temperature and pressure. While ingestion is the most common cause of gas embolism by hydrogen peroxide, cases have been reported where gas embolism was observed after skin contact with hydrogen peroxide in a surgical patient [10].

There are no standardized guidelines for the diagnosis and treatment of hydrogen peroxide ingestion and gas embolism. History is essential to identify the type of exposure and symptoms, and signs and symptoms such as abdominal pain, nausea, vomiting, and foaming at the mouth are suggestive of hydrogen peroxide ingestion. There is no consensus on the most appropriate diagnostic workup, however, endoscopy can be useful to evaluate for gastritis [6]. Although the plain film can identify portal venous gas embolism, the CT scan is the most frequently used imaging method [2]. In addition, no consensus exists regarding treatment. Most cases are managed conservatively. Gastrointestinal symptoms and mucosal irritation can be treated with H_2_ blockers or proton pump inhibitors, while in severe cases, where there is suspicion for airway compromise, endotracheal intubation may be warranted. Portal vein gas embolism is generally treated conservatively, however, hyperbaric oxygen has shown positive results in patients who ingested a concentrated solution [1-2]. Exposing patients to higher oxygen pressure facilitates the dissolution of gas into the blood system and the excess gas can be expelled via respiration. Our patient did not undergo such treatment and significantly improved in the following 48 hours. This is consistent with other previously reported 3% ingestion cases where patients are managed conservatively with positive outcomes (Table 1).

**Table 1:** Previously reported cases of portal vein gas embolism after ingestion of 3% hydrogen peroxide solution

Author	Age	Sex	Presenting symptoms	Ingested amount	Treatment	Clinical course
Arnsfield et al. [5]	21	M	Vomiting, Epigastric pain	One mouthful	Intubation for airway protection, Proton pump inhibitor, Antibiotics	Improved, Resolution of portal gas in repeat imaging in three days
Moon et al. [6]	25	F	Vomiting, Epigastric pain	40 cc	Oxygen, H2 blocker	Improved, Resolution of portal gas in repeat imaging in two days
Rackoff et al. [7]	2	M	Vomiting, Foaming around the mouth	Unknown	Oral antacids	Improved
Tanaka et al. [8]	73	M	Hematemesis, Epigastric pain	Unknown	Proton pump inhibitor	Improved

## Conclusions

To the best of our knowledge, this is the fifth case of gas embolism after 3% hydrogen peroxide ingestion, suggesting that a 3% solution can also lead to air embolism, and prompt treatment and monitoring is necessary. Furthermore, our patient’s clinical improvement with conservative management is in line with most of the previous reports and can reassure clinicians working in areas where hyperbaric treatment options are not available.
